# Long‐term exposure to air pollution and road traffic noise and incidence of dementia in the Danish Nurse Cohort

**DOI:** 10.1002/alz.13814

**Published:** 2024-05-08

**Authors:** Stéphane Tuffier, Jiawei Zhang, Marie Bergmann, Rina So, George Maria Napolitano, Thomas Cole‐Hunter, Matija Maric, Sonja Antic, Jørgen Brandt, Matthias Ketzel, Steffen Loft, Youn‐Hee Lim, Zorana Jovanovic Andersen

**Affiliations:** ^1^ Section of Environmental Health Department of Public Health University of Copenhagen Copenhagen Denmark; ^2^ Department of Epidemiology and Public Health University College London London UK; ^3^ Department of Neurology Aarhus University Hospital Aarhus Denmark; ^4^ The Research Clinic for Functional Disorders and Psychosomatics Aarhus University Hospital Aarhus Denmark; ^5^ Department of Environmental Science Aarhus University Roskilde Denmark; ^6^ iClimate Interdisciplinary Centre for Climate Change Aarhus University Roskilde Denmark; ^7^ Global Centre for Clean Air Research (GCARE) University of Surrey Guildford UK

**Keywords:** air pollution, cohort studies, dementia, Europe, incidence, noise transportation, nurses, physical activity, women

## Abstract

**INTRODUCTION:**

We examined the association of long‐term exposure to air pollution and road traffic noise with dementia incidence in the Danish Nurse Cohort.

**METHODS:**

Female nurses were followed for dementia incidence (hospital contact or medication prescription) from 1993/1999 to 2020. Air pollution and road traffic noise levels were estimated at nurses’ residences, and their associations with dementia were examined using Cox regression models.

**RESULTS:**

Of 25,233 nurses 1409 developed dementia. Particulate matter with a diameter of ≤2.5 µm (PM_2.5_) was associated with dementia incidence, after adjusting for lifestyle, socioeconomic status, and road traffic noise (hazard ratio [95% confidence interval] 1.35 [1.15–1.59] per interquartile range of 2.6 µg/m^3^). There was no association of PM2.5 with dementia in physically active nurses. Association with road traffic noise diminished after adjusting for PM₂.₅ (1.02 [0.93–1.11] per 7.6 dB).

**DISCUSSION:**

Long‐term exposure to air pollution increases risk of dementia, and physical activity may moderate this risk.

**Highlights:**

- Long‐term exposure to air pollution was associated with increased risk of dementia among female nurses from the Danish Nurse Cohort.
- Association of air pollution with dementia was independent of road traffic noise.
- Association of road traffic noise with dementia diminished after adjusting for air pollution.
- Physical activity moderated adverse effects of air pollution on dementia.

## 1 BACKGROUND

Dementia is a multifactorial condition characterized by a progressive memory loss and decline of cognitive functions. Alzheimer's disease (AD), vascular dementia (VD), and senile dementia are responsible for most dementia cases. Population aging together with an increase of non‐communicable disease and a lack of effective treatment for dementia, despite recent drug developments,^1^, ^2^ is expected to increase the burden of dementia: Worldwide prevalence cases are forecasted to increase from 60 million in 2019 to 150 million by 2050.^3^ Dementia's pathophysiology remains unclear, although deposits and plaques of amyloid beta and tau proteins, brain changes induced by vascular damage, and brain inflammation have been hypothesized to play a role in different types of dementia.^4^ Recently, environmental factors have been acknowledged as risk factors of dementia onset.^5^

Air pollution is the leading global environmental risk factor affecting everyone,^6^ and is linked to cardiovascular diseases, chronic and infectious respiratory diseases, type‐2 diabetes, and lung cancer, causing 6.7 million premature deaths globally every year.^7^ Air pollution ranks as the fourth leading risk factor for mortality and morbidity after tobacco, high blood pressure, and diet.^8^ The smallest fraction of airborne particles can enter blood circulation and pass through the blood‐brain barrier, as well as translocate along the olfactory nerve into the central nervous system, where they can trigger inflammation and oxidative stress, or produce direct toxic effects on the nervous system and the brain.^9^ Road traffic noise is another major environmental stressor causing annoyance, sleep disturbance, and stress^10^ that has been shown to increase the risk of cardiovascular disease^11^ and possibly psychiatric disorders.^12^ Air pollution and road traffic noise share both major sources (eg, road traffic) and biological pathways that can increase the risk of dementia including neuroinflammation or brain damage,^13^ but only a few epidemiological studies have considered these two exposures simultaneously.

**RESEARCH IN CONTEXT**

1. **Systematic review**: A literature review was performed using PubMed and a combination of MESH terms and keywords related to air pollution, noise, and dementia, and is summarized in Table S1 and Table S2. Evidence on the association between long‐term exposure to particulate matter with a diameter of ≤2.5 µm (PM_2.5_) and the risk of dementia is well established. For other air pollutants, evidence is less consistent and limited by the low number of studies. Evidence on road traffic noise is more limited, with only two out of five studies finding an association with the risk of dementia. Only two of these consider both air pollution and road traffic noise simultaneously.
2. **Interpretation**: This study shows that long‐term exposure to air pollution is strongly associated with incidence of dementia, independently of road traffic noise. We detected strong positive associations with PM_2.5_, PM_10_, NO_2_, and black carbon (BC), which were linear. We detected a weak positive association of long‐term exposure to road traffic noise and the incidence of dementia, which further weakened after adjustment for air pollutants. Physical activity as a lifestyle factor may moderate adverse effects of air pollution on dementia.
3. **Future directions**: More research is needed to identify groups that are most susceptible to harmful air pollution effects on dementia, for which preventive strategies can be designed. Disentangling individual effects of NO_2_, O_3_, BC, and road traffic noise is difficult due to shared sources, high correlation between them, and potential shared biological mechanisms. More research is needed to discern which are the responsible pollutants and sources of air pollution, in order to inform policy regulating air pollution.

The epidemiological evidence on long‐term exposure to air pollution and the risk of developing dementia has sharply increased in the last decade, with a number of studies showing an association (Table S1). The evidence is most consistent for particulate matter with a diameter of ≤2.5 µm (PM_2.5_), with recent meta‐analyses showing a 20% increase in the risk of developing dementia for each 5 µg/m^3^ increase.^14^ For other air pollutants, including particulate matter with a diameter of ≤10 µm (PM_10_), nitrogen dioxide (NO_2_), or ozone (O_3_), evidence is less consistent,^14^ whereas for black carbon (BC) there are very few studies.

Only five studies investigated associations between long‐term exposure to road traffic noise and the incidence of dementia (Table S2),^15^, ^16^, ^17^, ^18^, ^19^ presenting mixed results. Three studies found no association with road traffic noise.^15^, ^17^, ^19^ Carey et al. detected an association of road traffic noise with dementia, which diminished after adjustment for air pollution, indicating that the primary factor contributing to dementia risk might be air pollution rather than noise.^19^ Only two studies, Yu et al.’s small clinical study with detailed data on dementia diagnosis and other risk factors,^16^ and Cantuaria et al.’s nationwide Danish study including almost 2 million people,^18^ detected associations between road traffic noise and dementia, even after adjusting for air pollution. However, Cantuaria et al.’s^18^ nationwide analyses lacked data on individual lifestyle factors that may have confounded this association, and found a threshold relationship between road traffic noise and dementia with the association flattening out at levels above 65 dB.

Here we examine the association between long‐term exposure to both air pollution and road traffic noise and dementia incidence for up to 14 years in a cohort of female nurses in Denmark, for which we have previously documented associations of air pollution or road traffic noise^20^, ^21^ with cardiovascular^22^, ^23^, ^24^, ^25^ and metabolic diseases.^26^

## 2 METHODS

### 2.1 Data sources

#### 2.1.1 The Danish Nurse Cohort

The Danish Nurse Cohort (DNC)^27^ was designed to study the effects of hormone replacement therapy in menopausal women. All female nurses older than 44 years in 1993 were invited, regardless of their working status, via the Danish Nurse Organization (representing ≈95% of nurses in Denmark), and then again in 1999, when additional nurses who became eligible (turning 44) were also invited. A total of 28,731 nurses were included. Compared to the general Danish population, nurses in the cohort had healthier lifestyle habits, such as smoking less, being more physically active, but consuming more alcohol. Their use of health services was similar to the general population.^27^

The two surveys collected information about lifestyle factors (diet, smoking, alcohol consumption, and leisure‐time physical activity), socioeconomic status, working conditions, weight, height, and medical history including reproductive history and family history of cardiovascular disease and cancer.

For this study, we excluded nurses with pre‐existing dementia (at baseline, eg, 1993 or 1999), with missing information on covariates (at baseline) used in the fully adjusted model, or with missing exposure data for all the study period (between 1970 and 2020).

#### 2.1.2 Dementia definition

Using the Danish civil registration number (CPR), we linked nurses to the national Civil Registration^28^ to extract data on residential address history, date of death or emigration; to the Danish National Patient Registry (DNPR)^29^ and the Danish Psychiatric Central Register,^30^ to extract hospital contact data from 1978 until 2018; and to the Danish National Prescription Register^31^ to extract all medications prescribed in Denmark from 1995 until 2020. Dementia was defined as the occurrence of at least one hospital contact with primary diagnosis of dementia or one prescription of an anti‐dementia drug (Table S3). Previous studies showed good agreement and specificity between this register‐based definition and clinical dementia.^32^

### 2.2 Exposure

#### 2.2.1 Air pollution

Air pollution exposure was estimated using the Danish air pollution dispersion modeling system (DEHM/UBM/AirGIS).^33^, ^34^, ^35^, ^36^ In brief, DEHM/UBM/AirGIS is composed of three models: the Danish Eulerian Hemispheric Model (DEHM)^37^ provides regional background air pollution prediction (at 5.6 km^2^ resolution), the Danish Urban Background Model (UBM)^38^ provides local air pollution predictions (at 1 km^2^ resolution), and the Operational Street Pollution Model (OSPM) provides the residential address front door concentration. For the generation of the OSPM input parameters, a semiautomatic system has been constructed using the Geographic Information System (GIS) and available Danish registers (Road database, 3D building data). The modelling system has been extensively validated for PM₂.₅, PM₁₀, NO₂, O₃, and BC, and showed good agreement with a temporal correlation (*R*) of 0.45 to 0.96 and a spatial correlation of 0.32 to 0.92.^33^, ^39^

In this study, annual mean concentrations of PM₂․₅, PM₁₀, BC, NO₂, and O₃ were estimated at each nurse's residential address from 1979 until 2021. In case of relocation in a year, exposure was calculated as the weighted mean of monthly exposure at each address. Missing values were imputed using the last known value or the closest upcoming value if no prior data existed. Nurses without any available estimates were excluded.

#### 2.2.2 Road traffic noise

Road traffic noise was estimated using the Nord 2000 propagation model (DELTA 2001).^40^, ^41^ In brief, the model predicts noise from geocodes of location, street building geometry, road lines with information on yearly average daily traffic, traffic composition and speed, road type, and meteorology. Noise levels are calculated for the whole day (24 hours), and for the daytime (7:00 to 19:00), evening (19:00 to 22:00), and night hours (22:00 to 7:00). The equivalent A‐weighted sound pressure level (L_den_) was calculated by averaging day, evening, and night time levels with a 10‐dB penalty for night and 5‐dB for evening. Annual mean levels of L_den_ at each nurse's residence address were estimated from 1970 until 2014, and levels from 2015 to 2020 have been imputed using the last known value.

### 2.3 Statistical analysis

Cox proportional hazards models with age as the time scale were used to study the association of air pollutants (PM_2.5_, PM_10_, BC, NO_2_, and O_3_) and L_den_ (included as time‐varying variables) with the incidence of dementia. Nurses were followed from cohort baseline in 1993 or 1999 until the time of the first dementia diagnosis, death, emigration, or end of follow‐up on December 31, 2020, whichever came first.

We adjusted for relevant confounders in four different models: Model 1 accounted for the year of recruitment (eg, 1993 or 1999) using strata and was adjusted for the calendar year using a natural spline with 2 degrees of freedom, as both the outcome and exposure exhibited variation during the follow‐up^42^; Model 2 was additionally adjusted for body mass index (BMI), smoking, alcohol consumption, working and marital status, and family income (low: ≤127,000 Danish krone [DKK]; medium: 127,000 to 195,000 DKK; high: above 195,000 DKK); Model 3 was further adjusted for area (municipality)‐level socioeconomic status (SES) variables including municipality type (rural, provincial, or urban), median household wealth (ie, taxable assets), rate of unemployment, and rate of inhabitants receiving financial help from the municipality and of inhabitants with a high education; Model 4 (main model), was further adjusted for L_den_ or PM₂․₅ (in the L_den_ model). Confounding and mediation effects were identified on a directed acyclic graph (Figure S1). Proportional hazard assumption was checked using scaled Schoenfeld residual plots.

We ran models for PM₂․₅, PM₁₀, BC, NO₂, O₃, and L_den_ added as linear terms with time varying exposure windows of 14 years’ moving average, as development of dementia take decades. Exposure‐response functions for each pollutant was estimated using penalized smoothing splines with degrees of freedom from 2 to 5. The exposure‐response function with the lowest Akaike information criterion (AIC) was selected and tested for non‐linearity against the linear model with a likelihood‐ratio test. For each pair of exposure parameters with a Pearson's correlation coefficient below 0.7, we mutually added the second exposure to create a two‐pollutant model (based on Model 3).

Effect modification of the association between air pollution and dementia incidence by age, smoking status, BMI, physical activity, pre‐existing cardiovascular disease, shift work, marital status, and family income was assessed by adding a multiplicative term between the exposure and the effect modifier variable into Model 4. Physical activity was based on a self‐reported questionnaire on types of leisure‐time physical activities: reading, watching television, or engaging in other sedentary activity (low activity); going for a walk, riding a bicycle, or performing light physical activity (medium activity); or being an active athlete or performing heavy gardening, house work, and so forth for at least 4 hours per week, or vigorous training and participation in competitive sports several times a week (high activity).^43^ Significance of the effect modification term was tested using a likelihood‐ratio test.

Several sensitivity analysis were conducted: (1) associations with exposure windows of 1‐, 5‐ and 10‐years means pollutant levels were tested to assess sensitivity to exposure window definitions (Model 4); (2) analysis (Model 4) was repeated with dementia incidence defined only using hospitalization from the DNPR and by dementia subtype (ie, all hospitalizations, AD, or VD) to estimate associations with dementia subtype; (3) the analysis with December 31, 2018 as end of follow‐up was run to test whether results where sensitive to the lack of data from the DNPR after 2018; (4) as road traffic noise can be estimated below audible levels (35 dB), risk estimates and exposure‐response functions were tested after bounding noise levels to 35 dB; (5) analysis was repeated using daytime (L_day_) or nighttime (L_night_) noise levels; (6) dementia was alternatively defined in another sensitivity analysis, namely at least one hospital contact with a primary or secondary diagnosis of dementia or one prescription of anti‐dementia drug, whichever came first, and; (7) finally, models were compared with imputed pollutant levels and exposure windows of 1 year to models with non‐imputed levels to assess the robustness of the estimate associations after the imputation.

All analyses were performed with R 4.3.2,^44^ *tidyverse* ^45^ and *survival* ^46^ packages. Results are presented as hazard ratios (HRs) with 95% confidence intervals (CIs) per each interquartile range (IQR) increase of air pollutant or noise.

## 3 RESULTS

A total of 25,233 nurses were included in this study; 11 nurses were excluded because of prevalent dementia, 352 for missing exposure, and 2761 for missing covariates. From 1993 or 1999 until December 31, 2020, nurses were followed on average 23.36 years and a total of 575,877 person‐years, during which 1409 incident dementia cases were observed. Yearly incidence of dementia steadily increased from eight cases/year in 1993 to around 100 cases/year in 2014 and remained stable until the end of study. Median age of dementia onset was 79.1 years (Table 1). At baseline, nurses who developed dementia were older, more often single or widowed, retired, or residing in an urban area compared to nurses free of dementia during the follow‐up (Table 1). No differences were found regarding area SES characteristics and lifestyle, including smoking habits (for all three parameters—status, quantity, and duration).

**TABLE 1:** Characteristics of the nurses from the Danish Nurse Cohort at the cohort baseline in 1993 or 1999 by dementia incidence status at the end of follow‐up on December 31, 2020.

		Dementia	
	Total (*n* = 24,848)	Yes (*n* = 1409)	No (*n* = 23,439)
**Individual characteristics**			
**Inclusion year, *n* (%)**			
1993	16,340 (0.66)	1340 (0.95)	15,000 (0.64)
1999	8508 (0.34)	69 (0.05)	8439 (0.36)
**Age at inclusion, mean ± SD**	53.2 ± 8.11	60.75 ± 8.07	52.75 ± 7.88
**Body mass index, *n* (%)**			
Underweight (<18.5 kg/m^2^)	629 (0.03)	47 (0.03)	582 (0.02)
Normal (18.5–25 kg/m^2^)	17,151 (0.69)	944 (0.67)	16,207 (0.69)
Overweight (25–30 kg/m^2^)	5667 (0.23)	337 (0.24)	5330 (0.23)
Obese (>30 kg/m^2^)	1401 (0.06)	81 (0.06)	1320 (0.06)
**Smoking status, *n* (%)**			
Never	8536 (0.34)	452 (0.32)	8084 (0.34)
Previous	7647 (0.31)	482 (0.34)	7165 (0.31)
Current	8665 (0.35)	475 (0.34)	8190 (0.35)
**Smoking intensity (g/day), mean ± SD**	4.74 ± 8	4.23 ± 7.37	4.77 ± 8.03
**Smoking duration at baseline, mean ± SD**	10.24 ± 15.21	11.09 ± 16.78	10.19 ± 15.11
**Alcohol consumption, *n* (%)**			
Never (0 drinks per week)	3892 (0.16)	317 (0.22)	3575 (0.15)
Moderate (1–14 drinks per week)	15,296 (0.62)	797 (0.57)	14,499 (0.62)
Heavy (>15 drinks per week)	5660 (0.23)	295 (0.21)	5365 (0.23)
**Occupational status, *n* (%)**			
Working	19,427 (0.78)	714 (0.51)	18,713 (0.80)
Homeworker and other	673 (0.03)	23 (0.02)	650 (0.03)
Retired	4580 (0.18)	660 (0.47)	3920 (0.17)
Unemployed	168 (0.01)	12 (0.01)	156 (0.01)
**Marital status, *n* (%)**			
Married	17,491 (0.70)	848 (0.60)	16,643 (0.71)
Separated	409 (0.02)	19 (0.01)	390 (0.02)
Divorced	2803 (0.11)	160 (0.11)	2643 (0.11)
Single	2468 (0.10)	189 (0.13)	2279 (0.10)
Widow	1677 (0.07)	193 (0.14)	1484 (0.06)
**Leisure physical activity time at inclusion, *n* (%)**			
Low	1646 (0.07)	99 (0.07)	1547 (0.07)
Medium	16,398 (0.67)	985 (0.71)	15,413 (0.66)
High	6566 (0.27)	308 (0.22)	6258 (0.27)
**Family income in DKK, annual, mean ± SD**	170,888.73 ± 115,900.3	161,951.67 ± 142,073.24	171,425.97 ± 114,117.27
**Family income (categorical), *n* (%)**			
Low‐income (≤127,000 DKK)	5917 (0.24)	435 (0.31)	5482 (0.23)
Medium‐income (127,000–195,000 DKK)	12,443 (0.50)	699 (0.50)	11,744 (0.50)
High‐income (>195,000 DKK)	6488 (0.26)	275 (0.20)	6213 (0.27)
**CVD at inclusion, *n* (%)**	1128 (0.05)	60 (0.04)	1068 (0.05)
**Municipality characteristics**			
**Municipality type, *n* (%)**			
Rural	10,261 (0.41)	525 (0.37)	9736 (0.42)
Provincial	10,817 (0.44)	635 (0.45)	10,182 (0.43)
Urban	3770 (0.15)	249 (0.18)	3521 (0.15)
**Median wealth in DKK, mean ± SD**	151,011.25 ± 16,293.08	15,1391.93 ± 16551.6	150,988.36 ± 16,277.48
**Frequency of unemployment, mean ± SD**	2.07 ± 0.54	2.05 ± 0.53	2.07 ± 0.54
**Frequency of financial help, mean ± SD**	2.75 ± 1.26	2.78 ± 1.28	2.75 ± 1.26
**Frequency of high education, mean ± SD**	5.95 ± 4.81	6.13 ± 4.9	5.94 ± 4.81

Abbreviations: CVD, cardiovascular disease; DKK, Danish krone; SD, standard deviation.

**FIGURE 1 alz13814-fig-0001:**
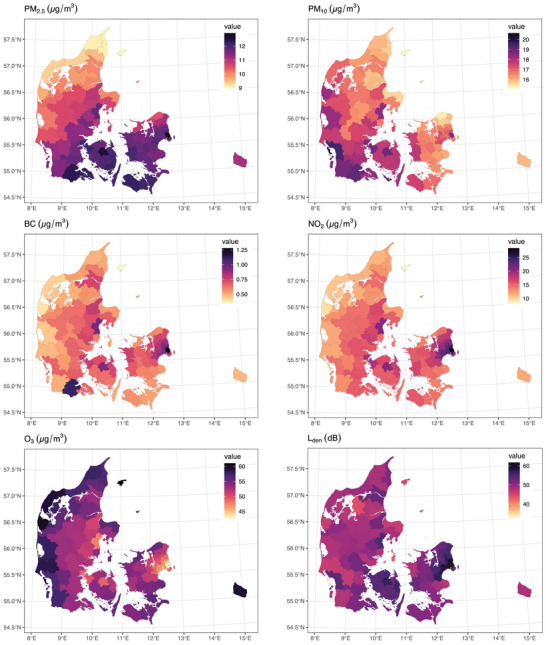
Levels of air pollution and road traffic noise levels of nurses from the Danish Nurse Cohort at the cohort baseline aggregated at the municipality level. BC, black carbon; L_den_, day–evening–night road traffic noise levels; NO₂, nitrogen dioxide; O₃, ozone; PM₂․₅, particulate matter with a diameter of ≤2.5 µm; PM₁₀, particulate matter with a diameter of ≤10 µm.

Air pollution and road traffic noise levels are detailed in Table 2. During the study period, all air pollutants levels decreased and O₃ concentration increased, whereas road traffic noise levels remained stable (Figure S2). PM₂.₅ and PM₁₀ were highly positively correlated, O₃ was negatively correlated with NO₂, while L_den_ was moderately correlated with BC, NO₂, O₃, and weakly correlated with PM₂.₅ or PM₁₀ (Figure S3). BC, NO₂, O₃ and L_den_ levels were higher in cities. PM₂.₅ concentrations were high in the southern parts of Denmark (Figure 1).

**TABLE 2:** Summary of air pollution and road traffic noise levels at residential addresses of nurses from the Danish Nurse Cohort from 1993 to 2020.

Exposure	Mean ± SD	Median (IQR)	Range (min–max)
**PM_2.5_ (µg/m^3^)**	10.73 ± 2.25	10.48 (2.96)	5.12–30.79
**PM_10_ (µg/m^3^)**	16.69 ± 2.57	16.47 (3.35)	9.93–42.4
**BC (µg/m^3^)**	0.69 ± 0.34	0.63 (0.32)	0.18–9.36
**NO_2_ (µg/m^3^)**	16.02 ± 6.35	14.75 (7.46)	4.46–74.14
**O_3_ (µg/m^3^)**	53.97 ± 5.15	54.63 (6.06)	13.13–68.69
**L_den_ (dB)**	53.31 ± 8.11	53.7 (9.5)	5.18–82.04

*Note*: Range minimum and maximum are calculated as the mean of the 5 lowest or highest individual values respectively for condidentialy reasons.

Abbreviations: BC, black carbon; IQR, interquartile range; L_den_, day–evening–night road traffic noise levels.; NO₂, nitrogen dioxide; O₃, ozone; PM₁₀, particulate matter with a diameter of ≤10 µm; PM₂․₅, particulate matter with a diameter of ≤2.5 µm; SD, standard deviation.

Long‐term exposure to all air pollutants, except O_3_, was associated with increased risk of dementia incidence. The HR (95% CI) values were 1.35 (1.15–1.59) per 2.61 µg/m^3^ for PM₂.₅ and 1.17 (1.07–1.27) per 6.18 µg/m^3^ for NO₂ after adjustment for SES, lifestyle, and municipality characteristics. Further adjustment for L_den_ did not change these associations, with an HR of 1.33 (1.11–1.59) for PM₂.₅ and 1.17 (1.06–1.30) for NO_2_ (Table 3). L_den_ showed weaker and statistically non‐significant association with incidence of dementia with an HR of 1.07 (0.99–1.16), which was further reduced to 1.02 (0.93–1.11) (Table 3) or 0.99 (0.90–1.09) per 7.64 dB (Table S4) when adjusting separately for PM₂.₅ or NO₂. PM_2.5_ association with dementia was also robust to adjustment for other air pollutants, whereas NO_2_ and BC associations were largely diminished after adjusting for PM₂.₅ (Table S4).

**TABLE 3:** Associations between 14 years’ mean exposure to air pollution or road traffic noise and incidence of dementia in the Danish Nurse Cohort.

	Model 1	Model 2	Model 3	Model 4
Exposure	HR (95% CI)	HR (95% CI)	HR (95% CI)	HR (95% CI)
**PM_2.5_ (µg/m^3^)**	1.35 (1.18–1.55)	1.32 (1.15–1.51)	1.35 (1.15–1.59)	1.33 (1.11–1.59)*
**PM_10_ (µg/m^3^)**	1.20 (1.07–1.34)	1.18 (1.06–1.33)	1.19 (1.06–1.35)	1.17 (1.02–1.34)*
**BC (µg/m^3^)**	1.09 (1.04–1.15)	1.08 (1.03–1.14)	1.09 (1.03–1.16)	1.08 (1.02–1.16)*
**NO_2_ (µg/m^3^)**	1.14 (1.07–1.21)	1.12 (1.05–1.19)	1.17 (1.07–1.27)	1.17 (1.06–1.30)*
**O_3_ (µg/m^3^)**	0.87 (0.82–0.93)	0.89 (0.83–0.95)	0.85 (0.78–0.93)	0.85 (0.77–0.94)*
**L_den_ (dB)**	1.10 (1.02–1.17)	1.08 (1.01–1.16)	1.07 (0.99–1.16)	1.02 (0.93–1.11)†

*Note*: HR were calculated using Cox proportional hazard models with 14 years’ average time varying exposure window. Model 1 is stratified for year of recruitment (eg., 1993 or 1999) and adjusted for calendar year (natural spline with 2 degree of freedom). Model 2 is additionally adjusted for individual factors (body mass index, smoking, alcohol consumption, working and martial status, and family income). Model 3 is further adjusted for area level covariates (municipality type [rural, provincial, or urban], median wealth, and frequencies of unemployment, inhabitants receiving financial aid, and high education). Model 4 is further adjusted for L_den_ (*) or PM₂․₅(†) in the L_den_ model. Nurses = 24,848, Dementia cases = 1409, Person‐years = 575,877. HR per IQR (PM₂․₅ = 2.96 PM₁₀ = 3.35; Black carbon = 0.32; NO₂ = 7.46; O₃ = 6.06; L_den _= 9.5).

Abbreviations: CI, confidence interval.; BC, black carbon; HR, hazard ratio; IQR, interquartile range; L_den_, day–evening–night road traffic noise levels; NO₂, nitrogen dioxide; O₃, ozone; PM₁₀, particulate matter with a diameter of ≤10 µm; PM₂․₅, particulate matter with a diameter of ≤2.5 µm.

**FIGURE 2 alz13814-fig-0002:**
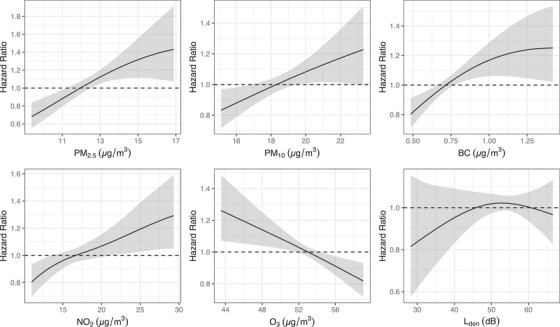
Exposure‐response functions of the associations between air pollution and road traffic noise and incidence of dementia in the Danish Nurse Cohort (*n* = 24,848). Exposure‐response functions were modeled using penalized spline. Reference value is the median of exposure levels. BC, black carbon; L_den_, day–evening–night road traffic noise levels; NO₂, nitrogen dioxide; O₃, ozone; PM₂․₅, particulate matter with a diameter of ≤2.5 µm; PM₁₀, particulate matter with a diameter of ≤10 µm.

**FIGURE 3 alz13814-fig-0003:**
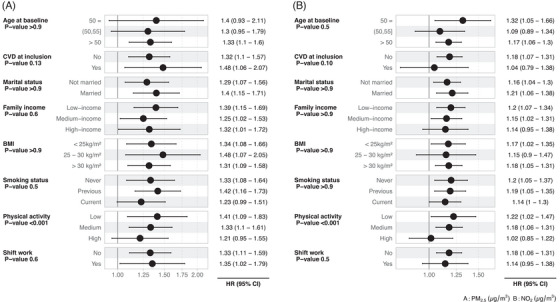
Effect modification analysis of associations between (A) PM₂․₅ and (B) NO_2_ and the incidence of dementia when considering interaction with characteristics of the nurses from the Danish Nurse Cohort at the cohort baseline in 1993 or 1999 (*n* = 24,848). Interaction was added as a multipliticative term in models adjusted for L_den_ (Models 4). Significance of an interaction term in the model was tested using the likelihood ratio test, and the *p*‐value reported in the figure. Interaction term estimates are provided in Table S8. HR values per each IQR of each air pollutant or noise are as follows: PM_2.5_ = 2.96; PM₁₀ = 3.35; black carbon = 0.32; NO₂ = 7.46; O₃ = 6.06; L_den _= 9.5. BMI, body mass index; CI, confidence interval; CVD, cardiovascular disease; HR, hazard ratio; PM₂․₅, particulate matter with a diameter of ≤2.5 µm; PM₁₀, particulate matter with a diameter of ≤10 µm

Exposure‐response functions of PM₂.₅, PM₁₀, NO₂, and L_den_ were slightly supra‐linear with no evidence for a safe threshold at low exposure (Figure 2). Reported level of physical activity was a significant effect modifier of PM₂.₅ and NO₂ association with dementia, with lower associations in nurses who reported high physical activity levels (Figure 3).

The study's main results remained consistent in sensitivity analyses. Models using either 1‐, 5‐, or 10‐year mean exposure windows yielded similar results to those with 14‐year windows (Table S5). When restricting the dementia incidence definition to hospitalization only, excluding medication prescription, associations with AD were slightly higher than for overall dementia, and no association existed with VD (Table S6). No differences in estimates were observed between models with or without bounding the L_den_ value levels to 35 dB; models using December 31, 2018 as the end of follow‐up; models using L_den_, L_day_, or L_night_; models with original and alternative dementia definitions (Table S7); and models with or without imputation of 1‐year noise exposure (not presented).

## 4 DISCUSSION

In this large cohort of Danish female nurses with detailed historical data on air pollution and road traffic noise, and over 20 years of follow‐up for dementia hospital contacts and medication prescriptions in the unique Danish nationwide registers, we found strong associations between long‐term exposure to major air pollutants and incidence of dementia. We found that these associations were independent of road traffic noise, whereas the weak association of road traffic noise with dementia diminished after adjusting for air pollution. We present the novel finding that physical activity may protect against adverse effects of air pollution on the risk of dementia.

Our study[Fig alz13814-fig-0001], [Fig alz13814-fig-0002], [Fig alz13814-fig-0003] results reinforce the established association between air pollution and dementia incidence and are consistent with those from previous studies. Specifically, the significant positive associations with PM_2.5_ and NO_2_, and negative association with O_3_, agree well with a recent meta‐analysis on long‐term exposure to air pollution and dementia incidence.^14^ For PM_2.5_, our estimate of 1.33 per 2.6 µg/m^3^ is substantially larger than the overall meta‐analysis estimate of 1.04 per 2 µg/m^3^.^14^ However, our estimate is consistent with Wilker et al.’s^14^ finding of higher estimates in European studies (1.21 per 2 µg/m^3^), as well as in studies with “active” case ascertainment, and with clinical diagnoses in smaller cohorts, as opposed to weak associations in studies with “passive” ascertainment from large administrative cohort studies based on hospital records. Our relatively strong association is likely explained by our lower misclassification bias in both exposure and outcome.^47^, ^48^ We used very fine, 14‐year historical, address‐specific air pollution data as well as accurate dementia diagnoses based on hospital contacts and medication from comprehensive Danish national registers. We found a strong association between NO₂ and dementia incidence, much stronger than that reported in Wilker et al. (HR of 1.24 vs 1.02 per 10 µg/m^3^),^14^ although consistent in size with several studies.^19^, ^49^, ^50^ Only four other studies estimated air pollution association with dementia incidence adjusted for road traffic noise,^15^, ^16^, ^17^, ^19^ and in line with our results, all found that associations with PM_2.5_ or NO_2_ (or NO_x_) persisted and were not attenuated after adjustment for road traffic noise. We present novel results of a strong association of BC and incidence of dementia with 1.29 (1.06–1.57) per 1 µg/m^3^, in line with a single other nationwide US study with data on BC.^51^ We found linear associations with PM_2.5_, NO_2_, and BC, showing that the association persists well below current EU standards of 25 and 40 µg/m3 for PM_2.5_ and NO_2_ respectively, as well as below World Health Organization guidelines of 5 and 10 µg/m^3^, respectively.^6^ The negative association with O_3_ is likely explained by the negative correlation of O_3_ with other traffic‐related air pollutants, as O_3_ is lower near sources of NO_2_ and BC, and increases further away from these sources. Thus, negative associations with O_3_ are likely explained by these local patterns, which have been captured by our air pollution prediction model, and not by a protective effect of O_3_, and is in line with other studies.^14^, ^16^

Two pollutant models show generally more robust associations for PM_2.5_ than for NO_2_ and BC, which were largely diminished after adjusting for PM₂.₅. However, due to large correlations between these pollutants, the results from two pollutant models should be interpreted with caution.

We find a slightly stronger association with AD than for overall dementia, and none with VD, in analyses limited to a subset of dementia incidence cases based on hospitalization for dementia, excluding medication prescription definitions. This was done as anti‐dementia medication is not specific to dementia subtype, and cannot be used to discern between different types of dementia. Moreover, VD hospitalization has been shown to have poor agreement with clinical diagnosis, limiting extrapolation of associations found.^32^

Lack of robust association between road traffic noise and incidence of dementia after adjustment for air pollution is in line with previous studies, where HR ranged from 1.02 to 1.16 per 10 dB.^16^, ^17^, ^18^, ^19^ Three out of four studies with data on both air pollution and road traffic noise found consistently that air pollution, and not road traffic noise, seemed to explain the association with dementia incidence,^15^, ^17^, ^19^ whereas Yu et al.^16^ suggest synergistic effects of the two exposures. In contrast to our study, in a nationwide Danish cohort, Cantuaria et al.^18^ found a strong association between exposure to road traffic noise and incidence of dementia which did not attenuate after adjusting for PM₂.₅ and NO₂, although they found a similar exposure‐response function showing no additional risk increase at noise levels above 50 db. Several factors may contribute to these differences. First, the DNC of our study is a selected and more homogeneous sample of the Danish population than that used by Cantuaria et al.^18^ This difference is reflected in the higher noise exposure of our sample compared to the whole Danish population, with 95% of the nurses’ exposure comprised between 47 and 67 dB, likely due to nurses living closer to urban areas or larger roads compared to the entire Danish population.^19^ Therefore, according to the similar exposure‐response function in our and Cantuaria's studies, we would expect nurses to show a lower association of road traffic noise with dementia incidence. Secondly, Cantuaria et al.^18^ used road traffic noise estimates for every 5 years (eg, 1995, 2000, 2005, 2010, and 2015) and linearly extrapolated missing years, whereas we used the NORD2000 model with finer time resolution of noise estimates, based on each year. Third, we were able to adjust for personal lifestyle factors such as smoking or alcohol consumption that can distort the observed association between noise and dementia, which were not available in nationwide cohort analyses by Cantuaria et al.^18^

Physical activity was the only factor modifying the association between air pollution and incidence of dementia, with nurses reporting high physical activity having lower risk of developing dementia when exposed to the same level of PM₂.₅ compared to nurses with low physical activity. Physical inactivity is recognized as a risk factor for dementia,^5^ whereas evidence of a possible interaction between physical activity and air pollution with respect to dementia is still very limited and mixed. A UK Biobank study found that physical activity measured using accelerometers was associated with a reduced risk of dementia, but not in participants with high exposure to air pollution including PM_2.5_.^52^ On the other hand, a Korean study found no interaction, showing a protective effect of physical activity on dementia regardless of air pollution exposure level.^53^ This finding needs to be confirmed in other studies, but still provides valuable insight for the development of preventive measures.

Strengths of this study include the use of a cohort previously used to study environmental exposure effects, with information on individual risk factors and behavior in combination with data from nationwide registers with over 20 years of follow‐up. Bias due to left truncation is likely to be minimal considering the age of nurses and the low prevalence of chronic diseases at baseline. Additionally, air pollution and road traffic noise exposure were assessed using high‐quality and detailed models, with individual yearly exposure levels for every nurse at their residential address starting 14 years before inclusion in the cohort (eg, a total of 35 years of continuous exposure data). Lastly, by including road traffic noise, we were able to adjust air pollution effects for noise exposure, although this could be improved by adding occupational, leisure, or commute‐related exposure as well as other types of noise such as aircraft noise.

The first limitation of this study is the use of registers to ascertain dementia cases as information about dementia subtype is not reliable enough^32^ and severity of dementia is not recorded, even though air pollution and road traffic noise may have different risks for some subtypes of dementia. However, previous validation studies have shown that for dementia cases in Danish registers the diagnoses are very specific and represent more severe cases of dementia.^32^ A second limitation is the inclusion of women only (by nature of our nurse cohort design) as they have a higher risk of developing AD,^54^, ^55^ but little is known on whether sex mediates the association between air pollution and the incidence of dementia.

## 5 CONCLUSION

In the DNC we show that long‐term exposure to air pollution can lead to the development of dementia, even after adjustment for road traffic noise. This brings strong new evidence that supports existing findings in the current literature, suggesting that air pollution is an important risk factor for dementia. Moreover, we present a novel finding that physical activity may help mitigate the adverse effects of air pollution on dementia. Finally, we find no evidence of an independent association between road traffic noise and dementia.

## CONFLICT OF INTEREST STATEMENT

The authors declare no competing financial interests or conflicts of interest. Author disclosures are available in the supporting information.

## CONSENT STATEMENT

This study was conducted in accordance with The Code of Ethics of the World Medical Association (Declaration of Helsinki) for experiments involving humans. The locally appointed ethics committee approved the research protocol and informed consent was obtained from the subjects (or their legally authorized representative). The authors thank all participants in the Danish Nurse Cohort (DNC) study and the DNC steering group study teams for their work and effort. DNC and Danish register data are protected by the Danish Data Protection Act and cannot be shared publicly. Access can be granted by Danish Data authorities (ie, Statistics Denmark).

## Supporting information

Supporting Information

Supporting Information
