# Supplementary material for: Long‐term exposure to air pollution and road traffic noise and incidence of dementia in the Danish Nurse Cohort
Source: Alzheimers Dement. 2024 May 8;20(6):4080–91. doi: 10.1002/alz.13814 (PMC11180848; doi:10.1002/alz.13814)
Supplement: Supplementary file 1 — Supporting Information [file ALZ-20-4080-s002.docx]

# Supplemental tables

## Table S1: Review of existing studies on association between long-term exposure to air pollution and incidence of dementia (HR presented per 5 μg/m³ for PM₂․₅_,_ 15 μg/m³ for PM₁₀; 10 μg/m³ for NO2; 1 μg/m³ for BC; 10 μg/m³ for O₃ and 10 db for L_den_).

| **Paper** | **Study area** | **Study period and follow up time** | **Population** | **Dementia source and definition** | **N total;  N case** | **Exposure assessment** | **Exposure** | **HR (95% CI)** |
| --- | --- | --- | --- | --- | --- | --- | --- | --- |
| **Andersson, 2018** [1] | Sweden | 1988-2010, 22 years | Betula cohort (random population sample from north of Sweden) | Clinical examination, DSM IV | 1,721; 302 | Air pollution: LUR, 50m^2^. Assigned to residential address at baseline, divided in quartiles. 2009-2010.  Noise: survey from the commune. Assigned to residential address at baseline.  Time: 2009-2010. | NO_x_ | < 9: 1  9–17: 1.14 (0.78–1.67)  17–26: 1.48 (1.03–2.12)  > 26: 1.41 (0.97–2.03) |
| **Carey, 2018** [2] | Greater London, UK | 2005-2013, 8 years | 50-79 years-old, with at least 1 year of registration in one of 75 practices | Register data, first dementia diagnosis | 130,978; 2,181 | Dispersion model based on meteorological data and data on source emissions (including road traffic). Assigned at postcodes address.  Time: 2004. | NO_2_ | 1.22 (1.07-1.38) |
|  |  |  |  |  |  |  | O_3_ | 0.75 (0.61-0.89) |
|  |  |  |  |  |  |  | PM_2.5_ | 1.43 (1.11-1.82) |
| **Cerza, 2019** [3] | Rome, Italy | 2001-2013, 12 years | ≥ 65 years-old in 2001, no prevalent dementia | Register data, first hospitalization | 350,844; 21,548 | LUR (ESCAPE). Assigned at residential address.  Time: 2001. | NO_2_ | 0.97 (0.96-0.99) |
|  |  |  |  |  |  |  | NO_x_ | 1 (1-1.01) |
|  |  |  |  |  |  |  | O_3_ | 1.06 (1.03-1.08) |
|  |  |  |  |  |  |  | PM_10_ | 1 (0.97-1.05) |
|  |  |  |  |  |  |  | PM_2.5_ | 0.99 (0.96-1.02) |
| **Chen, 2017** [4] | Ontario, Canada | 2001-2013, 12 years | 55-85 years-old, Ontario Population Health and Environment Cohort (ONPHEC) | Register data, one hospitalizations or three physician claims over 2 years or a prescription for dementia | 2,066,639; 257,816 | Satellite observation, atmospheric transport models and LUR. Assigned at postcode.  Time: 5 years moving average, 2y lag. | NO_2_ | 1.04 (1.03-1.05) |
|  |  |  |  |  |  |  | NO_2_ adjusted for O_3_ and PM_2.5_ | 1.03 (1.03-1.04) |
|  |  |  |  |  |  |  | O_3_ | 0.98 (0.97-0.99) |
|  |  |  |  |  |  |  | O_3_ adjusted for PM_2.5_ and NO_2_ | 0.99 (0.98-1.01) |
|  |  |  |  |  |  |  | PM_2.5_ | 1.04 (1.03-1.05) |
|  |  |  |  |  |  |  | PM_2.5_ adjusted for NO_2_ and O_3_ | 1.02 (1.01-1.03) |
| **Grande, 2020** [5] | Stockholm, Sweden | 2001-2013, 12 years | ≥60 years-old, resident from 2001 to 2004 (SNAC-K cohort) | Clinical examination, DSM IV | 2,927; 364 | Dispersion model based on local sources of emission. Estimated at residential address.  Time: 5 years moving average. | NO_x_ | 1.17 (1.01-1.36) |
|  |  |  |  |  |  |  | PM_2.5_ | 11.63 (5.05-26.48) |
| **Ilango, 2019** [6] | Ontario, Canada | 1996-2013, 17 years | ≥ 45 years-old, among participant to several national population (1996, 2000, 2003 and 2005), living for at least 5 years in Ontario. | Register data, one hospitalization or three physician claims over 2 years or a prescription for dementia | 34,391; 2,559 | Satellite data with global atmospheric chemistry transport model and geographically weighted regression (with land cover,  elevation and aerosol composition).  Time: 3 years moving average, from 1994. | NO_2_ | 1.11 (0.99-1.2) |
|  |  |  |  |  |  |  | PM_2.5_ | 1.14 (0.99-1.28) |
| **Jung, 2015** [7] | Taiwan | 2001-2010, 9 years | ≥ 65 years-old, sampled from Taiwan population | Register data, two diagnosis codes | 95,690; 1,399 | Monitoring data with IPW, assigned at postcode. From 2006-2010 PM_2.5_ estimated using PM_10_/PM_2.5_ ratio as PM_2.5_ was not available.  Time: 2001. | O_3_ | 1.03 (1-1.06) |
|  |  |  |  |  |  |  | PM_2.5_ | 1.01 (0.98-1.04) |
| **Kioumourtzoglou, 2016** [8] | 50 city in north east USA , USA | 1999-2010, 11 years | ≥ 65 years-old, from Medicare | Register data, first hospitalization | 9,817,806; 266,725 | Monitoring stations. Annual city averages.  Time: 1 years moving average. | PM_2.5_ | 1.47 (1.28-1.69) |
| **Lee, 2019** [9] | Southeastern, USA | 2000-2013, 13 years | ≥ 65 years-old, from Medicare fee-for-service | Register data, first hospitalization | 13,309,90; 1,409,599 | PM_2.5_ derived from satellite data and ground measurement. Annual average. Assigned at post code.  Time: 1 years moving average. | PM_2.5_ | 1.27 (1.26-1.28) |
| **Mortamais, 2021** [10] | 3 cities, France | 1990-2012, 22 years | ≥ 65 years-old resident, non-institutionalized | Clinical examination, DSM IV | 7,066; 791 | LUR (Elapse), back and forward extrapolated. Individual address of 2010.  Time: 10 years moving averages. | BC | 1.01 (0.98-1.04) |
|  |  |  |  |  |  |  | NO_2_ | 1.02 (0.9-116.64) |
|  |  |  |  |  |  |  | PM_2.5_ | 1.2 (1.08-1.32) |
| **Oudin, 2016** [11] | Umeå, Sweden | 1993-2010, 17 years | Betula cohort (random population sample from north of Sweden) | Clinical examination, DSM IV | 1,806; 302 | LUR 50m^2^, individual residence. Extrapolated from 2009-2010.  Time: 1 years moving average. | NO_x_ | 1.05 (0.98-1.12) |
| **Oudin, 2018** [12] | Umeå, Sweden | 1993-2010, 17 years | Betula cohort (random population sample from north of Sweden) | Clinical examination, DSM IV | 2,803; 302 | Wind and dispersion model from traffic exhaust.  Assigned at residential address.  Time: 1990, 2000 and 2010. | PM_2.5_ | 8.95 (1-81.3) |
| **Parra, 2022** [13] | UK | 2010-2016, 6 years | ≥ 60 years-old, UK Biobank | Register data, algorithm of the UK biobank | 187,194; 1,742 | LUR ESCAPE. Assigned at residential address.  Time: 2010. | NO_2_ | 1.19 (1.11-1.27) |
|  |  |  |  |  |  |  | NO_x_ | 1.07 (1.04-1.1) |
|  |  |  |  |  |  |  | PM_2.5_ | 1.87 (1.46-2.36) |
| **Ran, 2021** [14] | Hong-Kong | 1998-2011, 13 years | ≥ 65 years-old, residents at baseline | Register data, one hospitalization with I diagnosis of dementia | 59,349; 1,183 | Satellite derived PM_2.5_ with a surface extinction coefficient (no GEOS model). Assigned at residential address.  Time: 1 year moving average. | PM_2.5_ | 1.08 (1-1.17) |
| **Semmens, 2022** [15] | 4 cities; USA | 2000-2008, 8 years | ≥ 75 years-old, no neurologic trouble or dementia, no drugs (from GEMS RCT) | Clinical examination, DSM IV or Clinical Dementia Rating > 0.5 | 2,564; 326 | Spatiotemporal model.  Time: 20 year average before enrollment. | PM_2.5_ | 1.58 (1.13-2.2) |
| **Shaffer, 2021** [16] | Seattle, USA | 1994-2018, 24 years | ≥ 65 years-old, members of Kaiser insurance, randomly invited to participate in the cohort (Adult Changes in Thought). APOe status known. | Clinical examination, DSM IV | 4,166; 1,138 | LUR and geostatistical smoothing. Assigned to residential address.  Time: 10 years moving average. | PM_2.5_ | 2.1 (1.16-3.86) |
| **Shi, 2021** [17] | USA | 2000-2018, 18 years | ≥ 65years-old, nationwide medicare | Register data, diagnosis code of dementia | 12,233,371; 2,025,130 | Spatiotemporal models combining: satellite data, chemical transport model, LUR, meteorological data, EPA measurement. Averaged and assigned at ZIP codes.  Time: 5 years moving average. | NO_2_ | 1.02 (1.01-1.02) |
|  |  |  |  |  |  |  | O_3_ | 1 (44927) |
|  |  |  |  |  |  |  | PM_2.5_ | 1.1 (1.09-1.11) |
| **Smargiassi, 2020** [18] | Quebec, Canada | 2000-2012, 12 years | ≥ 65years-old, lived in the province for a least 4 years | Register data, one hospital admission or 3 health services used for dementia within 2 years or 1 prescription drug | 1,807,133; 199,826 | Satellite data with chemical transport model. Assigned to postcodes.  Time: 1 year moving average. | NO_2_ | 1.02 (1.01-1.02) |
|  |  |  |  |  |  |  | NO_2_ adjusted for PM_2.5_ | 1 (1-1.01) |
|  |  |  |  |  |  |  | PM_2.5_ | 1.07 (1.01-1.04) |
|  |  |  |  |  |  |  | PM_2.5_ adjusted for NO_2_ | 1.07 (1.05-1.08) |
| **Sullivan, 2021** [19] | Pennsylvania’s Allegheny County, North East, US | 2006-2014, 8 years | ≥ 65 years-old, sampled from registered voters | Clinical examination, Clinical Dementia Rating ≥1 | 1,572; 108 | EPA measurement, downscaled at census tract level with a Bayesian space-time model. Assigned at census tract level.  Time: 1 year moving average. | PM_2.5_ | 39.12 (8.33-249.14) |
| **Wang, 2022** [20] | USA | 2008-2018, 10 years | 74-92 years-old, women (WHIMS-ECHO cohort) | Clinical examination, dementia questionnaire (phone, validated for DSM IV) | 2,239; 398 | Kriging models based on EPA measurement. Assigned at residential address.  Time: 3y average before inclusion. | NO_2_ | 1.02 (0.89-1.14) |
|  |  |  |  |  |  |  | PM_2.5_ | 1.29 (0.97-1.74) |
| **Younan, 2021** [21] | USA | 1995-2010, 15 years | 65-80 years-old, women (WHIMS study) | Clinical examination, DSM IV and dementia questionnaire (phone, validated for DSM IV) | 6,485; 158 | Bayesian maximum entropy method with a chemical transport models including EPA ground measurements. Assigned at residential address.  Time: 1 year moving average. | PM_2.5_ | 1.39 (1.03-1.89) |
| **Yu, 2023** [22] | Sacramento, USA | 1998-2007, 9 years | ≥ 60 years-old, Mexican Americans | Clinical examination, DSM IV | 1,612; 104 | NO_2_, O_3_: LUR based on data from 2016, 30m^2^ and 1km^2^.  PM_2.5_: from 2004. Extrapolated using closest monitor station.  Noise: modelled from traffic data 2002.  All assigned at residence address.  Time: 2002. | NO_2_ | 1.08 (0.67-1.74) |
|  |  |  |  |  |  |  | NO_2_ adjusted for O_3_ | 1.05 (0.81-1.36) |
|  |  |  |  |  |  |  | NO_2_ adjusted for PM_2.5_ | 1 (0.76-1.28) |
|  |  |  |  |  |  |  | O_3_ | 1.02 (0.93-1.12) |
|  |  |  |  |  |  |  | O_3_ adjusted for NO_2_ | 1.02 (0.93-1.12) |
|  |  |  |  |  |  |  | O_3_ adjusted for PM_2.5_ | 1.01 (0.92-1.11) |
|  |  |  |  |  |  |  | PM_2.5_ | 2.42 (1-5.89) |
|  |  |  |  |  |  |  | PM_2.5_ adjusted for NO_2_ | 2.15 (0.91-5.2) |
|  |  |  |  |  |  |  | PM_2.5_ adjusted for O_3_ | 2.1 (0.91-4.92) |
| **Yuchi, 2020** [23] | Vancouver, Canada | 1994-2003, 9 years | 45-84 years-old, living in Vancouver from 1994-1998 | Register data, one diagnosis code or 3 physician claims over 2 year or a prescription | 633,949; 13,170 non AD | Air pollution: LUR.  Noise: noise modelled from traffic. Both assigned at residential address.  Time: 1994-1998 average. | BC | 1.01 (0.98-1.04) |
|  |  |  |  |  |  |  | NO_2_ | 1.01 (0.98-1.03) |
|  |  |  |  |  |  |  | PM_2.5_ | 1.07 (0.94-1.17) |

DSM IV: Diagnostic and Statistical Manual of Mental Disorders IVth edition ; LUR: Land use regression; PM₂․₅: particulate matter with a diameter ≤ 2.5 μm; PM₁₀: particulate matter with a diameter ≤ 10 μm;NO₂: nitrogen dioxide; O₃: ozone; L_den_: day–evening–night noise levels.

HR per 5 μg/m³ for PM₂․₅_,_ 15 μg/m³ for PM₁₀; 10 μg/m³ for NO2; 1 μg/m³ for BC; 10 μg/m³ for O₃ and 10 db for L_den_

## Table S2: Review of studied on association between long-term exposure to road traffic noise and incidence of dementia (HR presented per 5 μg/m³ for PM₂․₅_,_ 15 μg/m³ for PM₁₀; 10 μg/m³ for NO2; 1 μg/m³ for BC; 10 μg/m³ for O₃ and 10 db for L_den_).

| **Paper** | **Study area** | **Study period and follow up time** | **Population** | **Dementia source and definition** | **N total; N dementia case** | **Exposure assessment** | **Exposure** | **HR (95% CI)** |
| --- | --- | --- | --- | --- | --- | --- | --- | --- |
| **Andersson, 2018** [1] | Sweden | 1988-2010, 22 years | Betula cohort (random population sample from north of Sweden) | Clinical examination, DSM IV | 1,721; 302 | Air pollution: LUR, 50m^2^. Assigned to residential address at baseline, divided in quartiles. 2009-2010.  Noise: survey from the commune. Assigned to residential address at baseline.  Time: 2009-2010. | L_den_ | < 55 dB: 1  ≥ 55 dB: 0.97 (0.58–1.60) |
| **Cantuaria, 2021** [24] | Denmark | 2004-2017, 13 years | ≥ 60 years-old, nationwide | Register data, I or II diagnosis of dementia and/or one prescription for dementia | 1,938,994; 103,500 | Air pollution: AirGis model for 2000, 2010, 2015. Assigned at residential address. Noise: road traffic from Nord 2000 model for 1995, 2000, 2005, 2010, 2015, linearly extrapolated to missing years. Time: 10 years moving average. | L_den_ | <45 dB: 1 45-50 dB: 1.09 CI 1.06-1.12 50-55 dB: 1.16 CI 1.13-1.19 55-60 dB: 1.17 CI 1.14-1.20 60-65 dB: 1.16 CI 1.13-1.19 ≥65 dB: 1.16 CI 1.13-1.19 |
|  |  |  |  |  |  |  | L_den_ adjusted for PM_2.5_ and NO_2_ | <45 dB: 1  45–50 dB 1.08 (1.05 to 1.11) 50–55 dB: 1.15 (1.12 to 1.17) 55–60 dB: 1.14 (1.12 to 1.17) 60–65 dB: 1.12 (1.10 to 1.15) ≥65 dB: 1.04 (1.01 to 1.08) |
| **Carey, 2018** [2] | Greater London, UK | 2005-2013, 8years | 50-79 yo, with at least 1 year of registration in one of 75 practices | Register data, first dementia diagnosis | 130978; 2181 | Air pollution: Dispersion model based on meteorological data and data on source emissions (including road traffic). Noise: modelled from road traffic and land cover. All assigned at postcodes address. Time: 2004. | Lnight | 1.08 (1-1.21) |
|  |  |  |  |  |  |  | Lnight adjusted for NO_2_ | 1 (0.92-1.12) |
|  |  |  |  |  |  |  | NO_2_ adjusted Lnight | 1.22 (1.07-1.38) |
|  |  |  |  |  |  |  | PM_2.5_ adjusted for Lnight | 1.36 (1.05-1.9) |
| **Yu, 2023** [22] | Sacramento, US | 1998-2007, 9 years | ≥ 60 years-old, Mexican Americans | Clinical examination, DSM IV | 1,612; 104 | NO_2_, O_3_: LUR based on data from 2016, 30m^2^ and 1km^2^.  PM_2.5_: from 2004. Extrapolated using closest monitor station.  Noise: modelled from traffic data 2002.  All assigned at residence address.  Time: 2002. | L_den_ | 1.16 (0.92-1.44) |
|  |  |  |  |  |  |  | NO_2_ adjusted for L_den_ | 0.98 (0.75-1.28) |
|  |  |  |  |  |  |  | O_3_ adjusted for L_den_ | 1.02 (0.93-1.12) |
|  |  |  |  |  |  |  | PM_2.5_ adjusted for L_den_ | 2 (0.85-4.74) |
| **Yuchi, 2020** [23] | Vancouver, Canada | 1994-2003, 9 years | 45-84 years-old, living in Vancouver from 1994-1998 | Register data, one diagnosis code or 3 physician claims over 2 year or a prescription | 633,949; 13,170 non AD | Air pollution: LUR.  Noise: noise modelled from traffic.  All assigned at residential address.  Time: 1994-1998 average. | BC adjusted for L_den_ | 1.01 (0.98-1.04) |
|  |  |  |  |  |  |  | L_den_ | 1.02 (0.98-1.08) |
|  |  |  |  |  |  |  | NO_2_ adjusted for L_den_ | 1.01 (0.98-1.03) |
|  |  |  |  |  |  |  | PM_2.5_ adjusted for L_den_ | 1.07 (0.94-1.17) |

DSM IV: Diagnostic and Statistical Manual of Mental Disorders IVth edition: ; LUR: Land use regression; AD: Alzheimer's disease; PM₂․₅: particulate matter with a diameter ≤ 2.5 μm; PM₁₀: particulate matter with a diameter ≤ 10 μm; BC: black carbon;NO₂: nitrogen dioxide; O₃: ozone; L_den_: day–evening–night noise levels; L_night_: night noise levels.

HR per 5 μg/m³ for PM₂․₅_,_ 15 μg/m³ for PM₁₀; 10 μg/m³ for NO2; 1 μg/m³ for BC; 10 μg/m³ for O₃ and 10 db for L_den_

## Table S3: Dementia definition in the Danish registers.

|  | **Dementia type** | **Codes** |
| --- | --- | --- |
| **Danish National Patient Registry (from 1977),**  **Danish Psychiatric Central Register (from 1970)*** | AD | ICD-8: 290.10  ICD-10: F00.0, F00.1, F00.2 F00.9, G30.0, G30.1, G30.8, G30.9 |
|  | VaD | ICD-8: 293.0x, 293.1x  ICD-10: F01.0, F01.1, F01.2, F01.3, F01.8, F01.9 |
|  | Other dementia | ICD-8: 290.0x, 290.18, 290.19, 290.11  ICD-10: F02.0, F03, F02.8 + G31.8, G31.9 |
|  | All dementia | All the codes above |
| **Danish National Precription Registery (from 1995)** | All dementia | ATC: N06Dx |

Dementia was defined as the occurrence of at least one of the codes above; whichever came first.

*: ICD-8 until 1994 and ICD-10 from 1994. Only hospital contact with primary diagnosis.

AD: Alzheimer’s disease; VaD: Vascular dementia; ICD-8: International Classification of Disease 8^th^ revision; ICD-10: International Classification of Disease 10^th^ revision; ATC: Anatomical Therapeutic Chemical.

## Table S4: Associations between two exposure to air pollution and/or road traffic noise and incidence of dementia in the Danish Nurse Cohort.

| **Exposure** | **R***^*^* | **HR** | **95% CI** |
| --- | --- | --- | --- |
| **PM_2.5_ (μg/m^3^)** |  | 1.35 | (1.15-1.59) |
| + BC (μg/m^3^) | 0.63 | 1.31 | (1.01-1.69) |
| + NO_2_ (μg/m^3^) | 0.65 | 1.19 | (0.92-1.55) |
| + O_3_ (μg/m^3^) | −0.60 | 1.18 | (0.93-1.51) |
| + L_den_ (dB)† | 0.12 | 1.33 | (1.11-1.59) |
| **PM_10_ (μg/m^3^)** |  | 1.19 | (1.06-1.35) |
| + BC (μg/m^3^) | 0.66 | 1.08 | (0.89-1.32) |
| + NO_2_ (μg/m^3^) | 0.64 | 1.02 | (0.84-1.23) |
| + O_3_ (μg/m^3^) | −0.58 | 1.03 | (0.87-1.23) |
| + L_den_ (dB)† | 0.11 | 1.17 | (1.02-1.34) |
| **BC (μg/m^3^)** |  | 1.09 | (1.03-1.16) |
| + PM_2.5_ (μg/m^3^) | 0.63 | 1.02 | (0.93-1.11) |
| + PM_10_ (μg/m^3^) | 0.66 | 1.06 | (0.97-1.17) |
| + L_den_ (dB)† | 0.45 | 1.08 | (1.02-1.16) |
| **NO_2_ (μg/m^3^)** |  | 1.17 | (1.07-1.27) |
| + PM_2.5_ (μg/m^3^) | 0.65 | 1.09 | (0.95-1.25) |
| + PM_10_ (μg/m^3^) | 0.64 | 1.16 | (1.01-1.32) |
| + L_den_ (dB)† | 0.57 | 1.17 | (1.06-1.30) |
| **O_3_ (μg/m^3^)** |  | 0.85 | (0.78-0.93) |
| + PM_2.5_ (μg/m^3^) | −0.60 | 0.91 | (0.80-1.04) |
| + PM_10_ (μg/m^3^) | −0.58 | 0.87 | (0.76-0.98) |
| + L_den_ (dB)† | −0.52 | 0.85 | (0.77-0.94) |
| **L_den_ (dB)** |  | 1.07 | (0.99-1.16) |
| + PM_2.5_ (μg/m^3^)† | 0.12 | 1.02 | (0.93-1.11) |
| + PM_10_ (μg/m^3^) | 0.11 | 1.03 | (0.95-1.12) |
| + BC (μg/m^3^) | 0.45 | 1.03 | (0.94-1.12) |
| + NO_2_ (μg/m^3^) | 0.57 | 0.99 | (0.90-1.09) |
| + O_3_ (μg/m^3^) | −0.52 | 1.00 | (0.91-1.09) |

Two pollutants have been calculated using cox proportional hazard models for each pair of pollutants (indicated by “+”) with a Pearson correlation coefficient below 0.7. Exposure was 14 year’s average time varing exposure window.

Models were stratified for year of recruitment (eg. 1993 or 1999) and adjusted for calendar year (natural spline with 2 degree of freedom), individual factors (body mass index, smoking, alcohol consumption, working and martial status , and family income) and area levels covariates (municipality type (rural, provincial or urban), median wealth, frequency of unemployment, of inhabitants receiving financial, of high education) (Models 3).

Nurses= 24,848, Dementia cases=1,409, Person-years=575,877.

HR per IQR (PM₂․₅=2.96 PM₁₀=3.35; Black carbon=0.32; NO₂=7.46; O₃=6.06; L_den_=9.5).

^*^: Pearson correlation coefficient.

†: Models equivalent to Models 4 of Table 3.

PM₂․₅: particulate matter with a diameter ≤ 2.5 μm; PM₁₀: particulate matter with a diameter ≤ 10 μm; BC: black carbon; NO₂: nitrogen dioxide; O₃: ozone; L_den_: day–evening–night noise levels; HR: Hazard ratio; 95% CI: 95% confidence interval.

## Table S5: Associations between long-term exposure to air pollution or road traffic noise and incidence of dementia in the Danish Nurse Cohort with 5 years, 10 years or 14 year mean exposure.

| **Exposure** | **1 year mean** | **5 years mean** | **10 years mean** | **14 years mean** |
| --- | --- | --- | --- | --- |
|  | **HR (95% CI)** | **HR (95% CI)** | **HR (95% CI)** | **HR (95% CI)** |
| **PM_2.5_ (μg/m^3^)** | 1.23 (1.05-1.44) | 1.34 (1.10-1.62) | 1.32 (1.09-1.59) | 1.33 (1.11-1.59) |
| **PM_10_ (μg/m^3^)** | 1.13 (1.00-1.28) | 1.13 (0.99-1.30) | 1.15 (1.01-1.32) | 1.17 (1.02-1.34) |
| **BC (μg/m^3^)** | 1.10 (1.02-1.18) | 1.08 (1.01-1.16) | 1.08 (1.01-1.16) | 1.08 (1.02-1.16) |
| **NO_2_ (μg/m^3^)** | 1.18 (1.07-1.31) | 1.17 (1.05-1.30) | 1.17 (1.05-1.29) | 1.17 (1.06-1.30) |
| **O_3_ (μg/m^3^)** | 0.87 (0.79-0.96) | 0.84 (0.76-0.94) | 0.85 (0.77-0.94) | 0.85 (0.77-0.94) |
| **L_den_ (dB)** | 1.02 (0.95-1.10) | 1.01 (0.93-1.09) | 1.02 (0.94-1.11) | 1.02 (0.93-1.11) |

HR were calculated using cox proportional hazard models using 1 year, 5 years, 10 years or14 years (models 4) time varing exposure windows.

Models stratified for year of recruitment (eg. 1993 or 1999) and adjusted for calendar year (natural spline with 2 degree of freedom), individual factors (body mass index, smoking, alcohol consumption, working and martial status , and family income) and area levels covariates (municipality type (rural, provincial or urban), median wealth, frequency of unemployment, of inhabitants receiving financial, of high education) and mutually adjusted for noise (L_den_) or PM₂․₅ (Models 4).

Nurses= 24,848, Dementia cases=1,409, Person-years=575,877.

HR per IQR (PM₂․₅=2.96 PM₁₀=3.35; Black carbon=0.32; NO₂=7.46; O₃=6.06; L_den_=9.5).

PM₂․₅: particulate matter with a diameter ≤ 2.5 μm; PM₁₀: particulate matter with a diameter ≤ 10 μm; BC: black carbon; NO₂: nitrogen dioxide; O₃: ozone; L_den_: day–evening–night noise levels; HR: Hazard ratio; 95% CI: 95% confidence interval.

## Table S6: Associations between long-term exposure to air pollution and noise and hospitalization for all type of dementia, Alzheimer’ disease or vascular dementia in the Danish Nurse Cohort.

| **Exposure** | **All hospitalizations** 576,661 person-years 1,079 cases | **Alzheimer’s disease** 578,676 person-years 692 cases | **Vascular dementia** 581,184 person-years 168 cases |
| --- | --- | --- | --- |
|  | **HR (95% CI)** | **HR (95% CI)** | **HR (95% CI)** |
| **PM_2.5_ (μg/m^3^)** | 1.24 (1.02-1.51) | 1.38 (1.06-1.79) | 1.09 (0.67-1.76) |
| **PM_10_ (μg/m^3^)** | 1.12 (0.97-1.31) | 1.27 (1.05-1.54) | 1.15 (0.80-1.66) |
| **BC (μg/m^3^)** | 1.07 (1.00-1.15) | 1.10 (1.01-1.20) | 0.94 (0.77-1.15) |
| **NO_2_ (μg/m^3^)** | 1.12 (1.00-1.26) | 1.19 (1.03-1.38) | 0.99 (0.73-1.33) |
| **O_3_ (μg/m^3^)** | 0.91 (0.81-1.02) | 0.85 (0.74-0.98) | 1.02 (0.76-1.36) |
| **L_den_ (dB)** | 0.99 (0.90-1.09) | 1.00 (0.88-1.13) | 1.02 (0.80-1.32) |

Alzheimer’ disease or vascular dementia were defined using hospitalization code (Table S3). HR were calculated using cox proportional hazard models and 14 years time varing exposure window.

Models stratified for year of recruitment (eg. 1993 or 1999) and adjusted for calendar year (natural spline with 2 degree of freedom), individual factors (body mass index, smoking, alcohol consumption, working and martial status , and family income) and area levels covariates (municipality type (rural, provincial or urban), median wealth, frequency of unemployment, of inhabitants receiving financial, of high education) and mutually adjusted for noise (L_den_) or PM₂․₅ (Models 4).

HR per IQR (PM₂․₅=2.96 PM₁₀=3.35; Black carbon=0.32; NO₂=7.46; O₃=6.06; L_den_=9.5).

PM₂․₅: particulate matter with a diameter ≤ 2.5 μm; PM₁₀: particulate matter with a diameter ≤ 10 μm; BC: black carbon; NO₂: nitrogen dioxide; O₃: ozone; L_den_: day–evening–night noise levels; HR: Hazard ratio; 95% CI: 95% confidence interval.

## Table S7: Sensitivity analysis of the associations between long-term exposure to air pollution and incidence of dementia.

| **Exposure** |  | **5 years mean** | **14 years mean** |
| --- | --- | --- | --- |
|  |  | **HR (95% CI)** | **HR (95% CI)** |
| 1. **Bounded L_den_***† | | | |
| **L_den_ (dB)** | No bound | 1.05 (0.98-1.14) | 1.07 (0.99-1.16) |
|  | Bound to 35 db | 1.05 (0.97-1.14) | 1.07 (0.99-1.16) |
| 1. **End of follow-up on the 31th Dec 2018*‡** | | | |
| **PM_2.5_ (μg/m^3^)** |  | 1.26 (1.04-1.52) | 1.27 (1.07-1.50) |
| **PM_10_ (μg/m^3^)** |  | 1.11 (0.97-1.27) | 1.15 (1.01-1.31) |
| **BC (μg/m^3^)** |  | 1.08 (1.01-1.15) | 1.08 (1.02-1.15) |
| **NO_2_ (μg/m^3^)** |  | 1.13 (1.03-1.24) | 1.14 (1.04-1.25) |
| **O_3_ (μg/m^3^)** |  | 0.88 (0.80-0.96) | 0.88 (0.80-0.96) |
| **L_den_ (dB)** |  | 1.05 (0.97-1.14) | 1.07 (0.99-1.17) |
| 1. **Dementia definition including secondary diagnosis*§** | | | |
| **PM_2.5_ (μg/m^3^)** |  | 1.37 (1.16-1.61) | 1.37 (1.18-1.59) |
| **PM_10_ (μg/m^3^)** |  | 1.14 (1.01-1.28) | 1.18 (1.05-1.32) |
| **BC (μg/m^3^)** |  | 1.09 (1.03-1.15) | 1.09 (1.04-1.15) |
| **NO_2_ (μg/m^3^)** |  | 1.15 (1.07-1.25) | 1.17 (1.08-1.26) |
| **O_3_ (μg/m^3^)** |  | 0.86 (0.79-0.93) | 0.85 (0.79-0.92) |
| **L_den_ (dB)** |  | 1.05 (0.98-1.13) | 1.08 (1.00-1.16) |
| 1. **Analysis using daytime (L_day_)** **and nighttime noise (L_night_)*** † | | | |
| **L_day_ (dB)** |  | 1.06 (0.98-1.14) | 1.08 (1.00-1.16) |
| **L_night_ (dB)** |  | 1.05 (0.97-1.13) | 1.07 (0.99-1.16) |

HR were calculated using cox proportional hazard models with 5 or 14 year’s average time varying exposure window. 1: road traffic noise levels were bound to 35db, which is the lowest sound intensity audible to the human ear. 2: end of follow date identical to the most recent avaible data from the National Patient register (hospitalisation contact only).

*: Models were stratified for year of recruitment (eg. 1993 or 1999) and adjusted for calendar year (natural spline with 2 degree of freedom), individual factors (body mass index, smoking, alcohol consumption, working and martial status , and family income) and area levels covariates (municipality type (rural, provincial or urban), median wealth, frequency of unemployment, of inhabitants receiving financial, of high education) (Models 3).

†: 575,877 person-years, 1,409 cases

‡: 539,347 person-years, 1,243 cases

§: 574998 person-years, 1,612.00 cases

HR per IQR (PM₂․₅=2.96 PM₁₀=3.35; Black carbon=0.32; NO₂=7.46; O₃=6.06; L_den_=9.5

; L_day_=9.5; L_night_=9.5).

PM₂․₅: particulate matter with a diameter ≤ 2.5 μm; PM₁₀: particulate matter with a diameter ≤ 10 μm; BC: black carbon; NO₂: nitrogen dioxide; O₃: ozone; L_den_: day–evening–night noise levels; L_day_: daytime noise; L_night_: nighttime noise; HR: Hazard ratio; 95% CI: 95% confidence interval.

## Table S8: Estimates for interaction term of associations between PM2.5 and NO2 and incidence of dementia when considering interaction with characteristics of the nurses from the Danish Nurse Cohort at the cohort baseline in 1993 or 1999 (n=24,848).

|  |  | **PM_2.5_ (μg/m^3^)** | | **NO_2_ (μg/m^3^)** |
| --- | --- | --- | --- | --- |
|  |  | **HR (95% CI)** | | **HR (95% CI)** |
| **Age at baseline** | 50 ≤ | Ref | | Ref |
|  | (50,55] | 0.93 (0.62-1.40) | | 0.83 (0.63-1.09) |
|  | > 50 | 0.95 (0.63-1.43) | | 0.89 (0.71-1.11) |
| **CVD at inclusion** | No | Ref | | Ref |
|  | Yes | 1.13 (0.83-1.53) | | 0.88 (0.67-1.16) |
| **Marital status** | Not married | Ref | | Ref |
|  | Married | 1.09 (0.93-1.26) | | 1.04 (0.92-1.18) |
| **Family income** | Low-income | Ref | | Ref |
|  | Medium-income | 0.90 (0.76-1.05) | | 0.96 (0.85-1.09) |
|  | High-income | 0.94 (0.74-1.20) | | 0.96 (0.79-1.15) |
| **BMI** | < 25kg/m² | 0.89 (0.66-1.19) | | 1.02 (0.80-1.30) |
|  | 25 - 30 kg/m² | 0.90 (0.66-1.24) | | 1.02 (0.79-1.32) |
|  | > 30 kg/m² | Ref | | Ref |
| **Smoking status** | Never | Ref | | Ref |
|  | Previous | 1.06 (0.89-1.27) | | 0.99 (0.86-1.14) |
|  | Current | 0.92 (0.77-1.11) | | 0.95 (0.82-1.10) |
| **Physical activity** | Low | Ref | | Ref |
|  | Medium | 0.94 (0.75-1.18) | 0.96 (0.80-1.16) | |
|  | High | 0.86 (0.65-1.13) | 0.83 (0.66-1.04) | |
| **Shift work** | No | Ref | Ref | |
|  | Yes | 1.02 (0.80-1.30) | 0.97 (0.81-1.16) | |

Interaction was added as a multipliticative term in models adjusted for L_den_ (Models 4). PM₂․₅: particulate matter with a diameter ≤ 2.5 μm; NO₂: nitrogen dioxide; BMI: body mass index; HR: Hazard ratio; 95% CI: 95% confidence interval. Interaction term estimates are provided in Supplemental Table 8.

HR per IQR (PM₂․₅=2.96 PM₁₀=3.35; Black carbon=0.32; NO₂=7.46; O₃=6.06; L_den_=9.5).

# Supplemental figures

## Figure S1: Directed acyclic graph of relations between air pollution, road traffic noise and incidence of dementia.


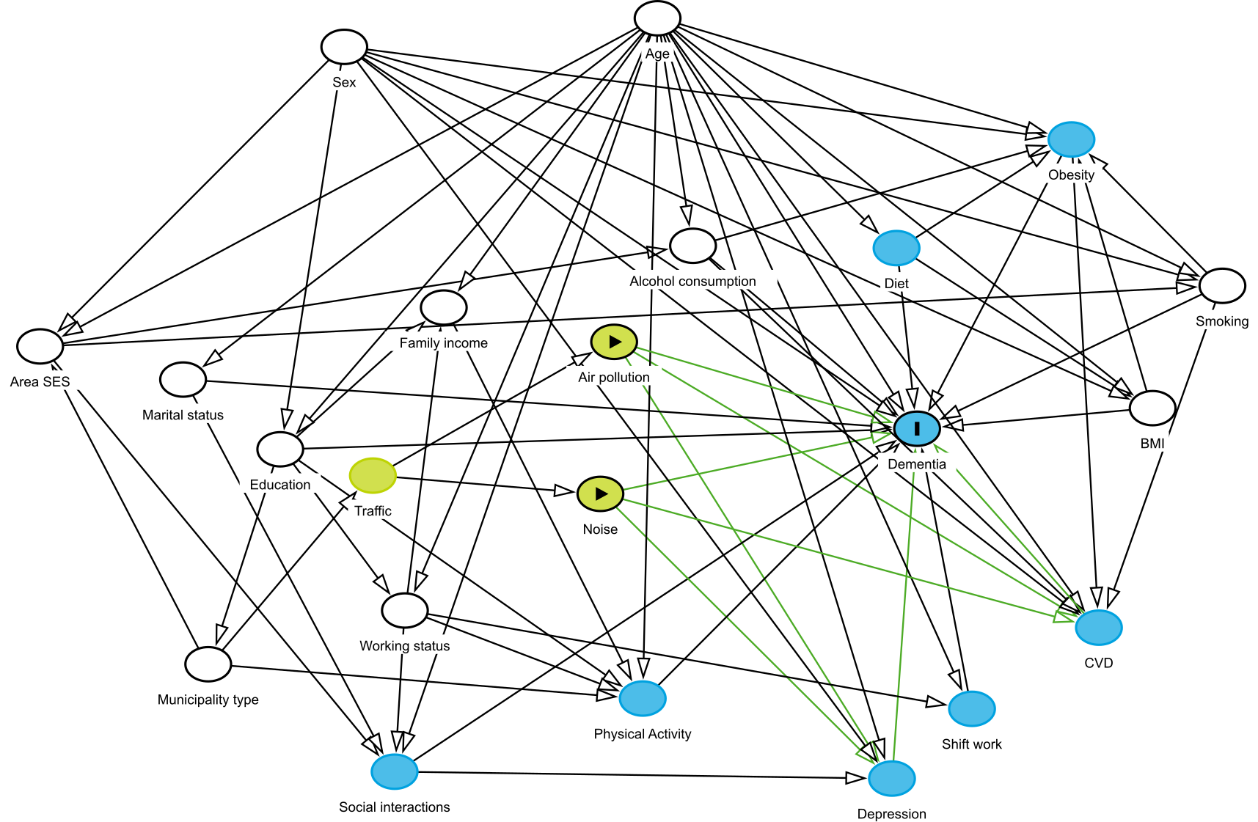


SES: social econononics status; BMI: body mass index; CVD: Cardiovascular disease

## Figure S2: Trends in air pollution and road traffic noise levels at residential address of nurses from the Danish Nurse Cohort from 1993 until 2020.


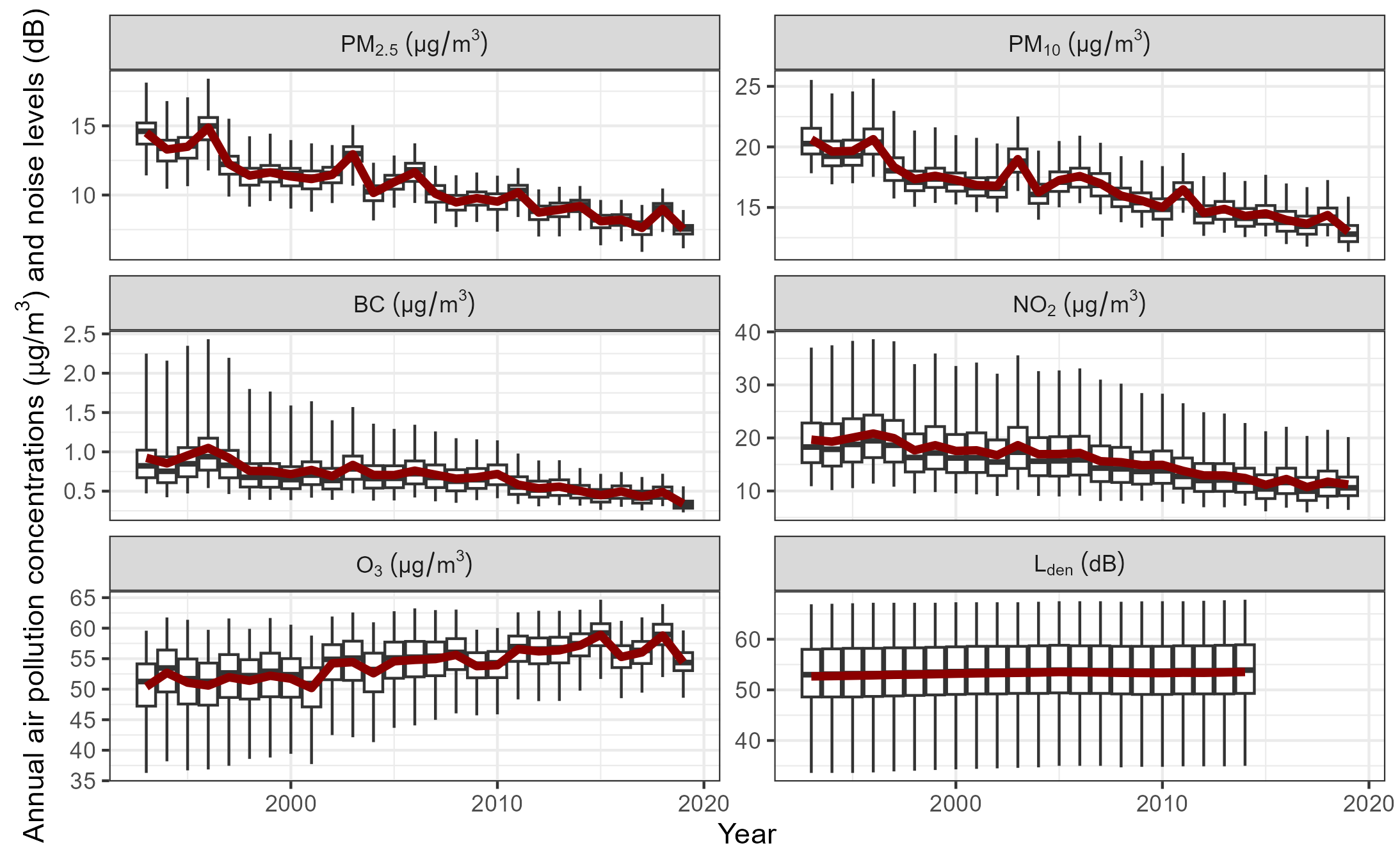


Red line represent annual average trend. Boxplots indicate 2.5, 25, 50, 75 and 97.5 quantiles. Missing noise values after 2014 have been imputed using the last know value for each nurse.

PM₂․₅: particulate matter with a diameter ≤ 2.5 μm; PM₁₀: particulate matter with a diameter ≤ 10 μm; BC: black carbon;NO₂: nitrogen dioxide; O₃: ozone; L_den_: day–evening–night noise levels.

## Figure S3: Correlation between air pollutants and road traffic noise at the Danish Nurse Cohort baseline in 1993 or 1999.


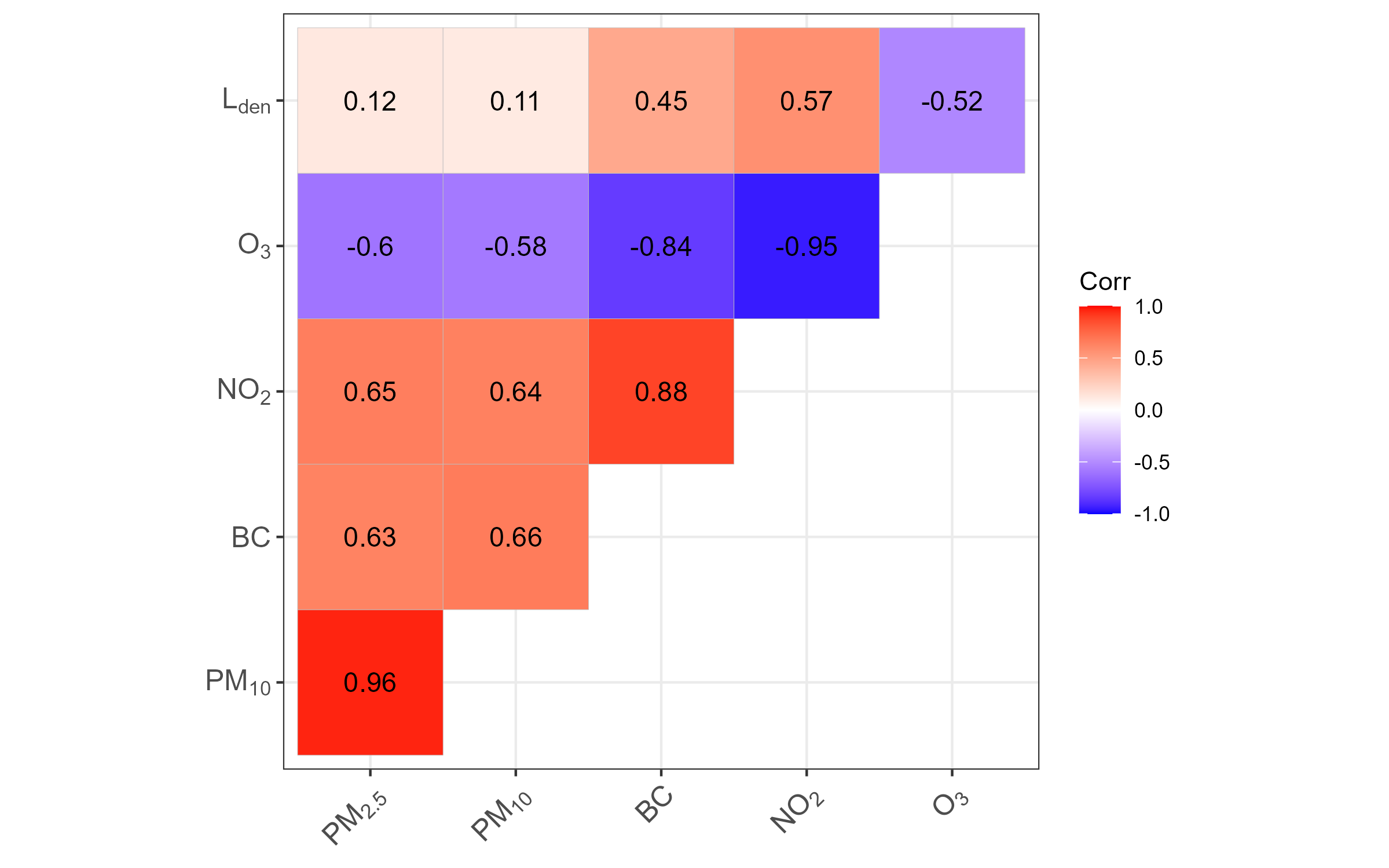


Pearson correlation coefficient was used.

PM₂․₅: particulate matter with a diameter ≤ 2.5 μm; PM₁₀: particulate matter with a diameter ≤ 10 μm; BC: black carbon; NO₂: nitrogen dioxide; O₃: ozone; L_den_: day–evening–night noise levels.

# PubMed search strategy

Details of the PubMed search resulting in Table S1 and S2

(

(Environmental Pollution[MeSH Major Topic]) OR

(

(

(air[Title/Abstract] AND pollution[Title/Abstract]) OR

(

(particulate matter[Title/Abstract]) OR

PM[Title/Abstract] OR

(nitrogen dioxide[Title/Abstract]) OR nitrates[Title/Abstract] OR

NOx[Title/Abstract] OR NO2[Title/Abstract] OR NO3[Title/Abstract] OR

ozone[Title/Abstract] OR

o3[Title/Abstract] OR

BC[Title/Abstract]

)

) OR

("road*"[Title/Abstract] NOT "roadmap*"[Title/Abstract]) OR "Traffic"[Title/Abstract] OR "diesel"[Title/Abstract] OR

(Noise[Title/Abstract])

)

)

AND

(

Brain Diseases[MeSH] OR

(

(dementia[Title/Abstract]) OR

(alzheimer[Title/Abstract]) OR

(pyschiatric*[Title/Abstract]) OR

(cogniti*[Title/Abstract])

)

)

# References

[1] Andersson J, Oudin A, Sundström A, Forsberg B, Adolfsson R, Nordin M. Road traffic noise, air pollution, and risk of dementia - results from the Betula project. Environ Res 2018;166:334–9. https://doi.org/10.1016/j.envres.2018.06.008.

[2] Carey IM, Anderson HR, Atkinson RW, Beevers SD, Cook DG, Strachan DP, et al. Are noise and air pollution related to the incidence of dementia? A cohort study in London, England. BMJ Open 2018;8:e022404. https://doi.org/10.1136/bmjopen-2018-022404.

[3] Cerza F, Renzi M, Gariazzo C, Davoli M, Michelozzi P, Forastiere F, et al. Long-term exposure to air pollution and hospitalization for dementia in the Rome longitudinal study. Environ Health Glob Access Sci Source 2019;18:72. https://doi.org/10.1186/s12940-019-0511-5.

[4] Chen H, Kwong JC, Copes R, Hystad P, van Donkelaar A, Tu K, et al. Exposure to ambient air pollution and the incidence of dementia: A population-based cohort study. Environ Int 2017;108:271–7. https://doi.org/10.1016/j.envint.2017.08.020.

[5] Grande G, Ljungman PLS, Eneroth K, Bellander T, Rizzuto D. Association Between Cardiovascular Disease and Long-term Exposure to Air Pollution With the Risk of Dementia. JAMA Neurol 2020;77:801–9. https://doi.org/10.1001/jamaneurol.2019.4914.

[6] Ilango SD, Chen H, Hystad P, van Donkelaar A, Kwong JC, Tu K, et al. The role of cardiovascular disease in the relationship between air pollution and incident dementia: a population-based cohort study. Int J Epidemiol 2020;49:36–44. https://doi.org/10.1093/ije/dyz154.

[7] Jung C-R, Lin Y-T, Hwang B-F. Ozone, Particulate Matter, and Newly Diagnosed Alzheimer’s Disease: A Population-Based Cohort Study in Taiwan. J Alzheimers Dis 2015;44:573–84. https://doi.org/10.3233/JAD-140855.

[8] Kioumourtzoglou M-A, Spiegelman D, Szpiro AA, Sheppard L, Kaufman JD, Yanosky JD, et al. Exposure measurement error in PM2.5 health effects studies: A pooled analysis of eight personal exposure validation studies. Environ Health 2014;13:2. https://doi.org/10.1186/1476-069X-13-2.

[9] Lee M, Schwartz J, Wang Y, Dominici F, Zanobetti A. Long-term effect of fine particulate matter on hospitalization with dementia. Environ Pollut 2019;254:112926. https://doi.org/10.1016/j.envpol.2019.07.094.

[10] Mortamais M, Gutierrez L-A, de Hoogh K, Chen J, Vienneau D, Carrière I, et al. Long-term exposure to ambient air pollution and risk of dementia: Results of the prospective Three-City Study. Environ Int 2021;148:106376. https://doi.org/10.1016/j.envint.2020.106376.

[11] Oudin A, Forsberg B, Adolfsson AN, Lind N, Modig L, Nordin M, et al. Traffic-Related Air Pollution and Dementia Incidence in Northern Sweden: A Longitudinal Study. Environ Health Perspect 2016;124:306–12. https://doi.org/10.1289/ehp.1408322.

[12] Oudin A, Segersson D, Adolfsson R, Forsberg B. Association between air pollution from residential wood burning and dementia incidence in a longitudinal study in Northern Sweden. PLOS ONE 2018;13:e0198283. https://doi.org/10.1371/journal.pone.0198283.

[13] Parra KL, Alexander GE, Raichlen DA, Klimentidis YC, Furlong MA. Exposure to air pollution and risk of incident dementia in the UK Biobank. Environ Res 2022;209. https://doi.org/10.1016/j.envres.2022.112895.

[14] Ran J, Schooling CM, Han L, Sun S, Zhao S, Zhang X, et al. Long-term exposure to fine particulate matter and dementia incidence: A cohort study in Hong Kong. Environ Pollut 2021;271:116303. https://doi.org/10.1016/j.envpol.2020.116303.

[15] Semmens EO, Leary CS, Fitzpatrick AL, Ilango SD, Park C, Adam CE, et al. Air pollution and dementia in older adults in the Ginkgo Evaluation of Memory Study. Alzheimers Dement 2023;19:549–59. https://doi.org/10.1002/alz.12654.

[16] Shaffer RM, Blanco MN, Li G, Adar SD, Carone M, Szpiro AA, et al. Fine Particulate Matter and Dementia Incidence in the Adult Changes in Thought Study. Environ Health Perspect 2021;129:87001. https://doi.org/10.1289/EHP9018.

[17] Shi L, Steenland K, Li H, Liu P, Zhang Y, Lyles RH, et al. A national cohort study (2000-2018) of long-term air pollution exposure and incident dementia in older adults in the United States. Nat Commun 2021;12:6754. https://doi.org/10.1038/s41467-021-27049-2.

[18] Smargiassi A, Sidi EAL, Robert L-E, Plante C, Haddad M, Gamache P, et al. Exposure to ambient air pollutants and the onset of dementia in Québec, Canada. Environ Res 2020;190:109870. https://doi.org/10.1016/j.envres.2020.109870.

[19] Sullivan KJ, Ran X, Wu F, Chang C-CH, Sharma R, Jacobsen E, et al. Ambient fine particulate matter exposure and incident mild cognitive impairment and dementia. J Am Geriatr Soc 2021;69:2185–94. https://doi.org/10.1111/jgs.17188.

[20] Wang X, Younan D, Millstein J, Petkus AJ, Garcia E, Beavers DP, et al. Association of improved air quality with lower dementia risk in older women. Proc Natl Acad Sci 2022;119:e2107833119. https://doi.org/10.1073/pnas.2107833119.

[21] Younan D, Wang X, Gruenewald T, Gatz M, Serre ML, Vizuete W, et al. Racial/Ethnic Disparities in Alzheimer’s Disease Risk: Role of Exposure to Ambient Fine Particles. J Gerontol Ser A 2022;77:977–85. https://doi.org/10.1093/gerona/glab231.

[22] Yu Y, Su J, Jerrett M, Paul KC, Lee E, Shih I-F, et al. Air pollution and traffic noise interact to affect cognitive health in older Mexican Americans. Environ Int 2023;173:107810. https://doi.org/10.1016/j.envint.2023.107810.

[23] Yuchi W, Sbihi H, Davies H, Tamburic L, Brauer M. Road proximity, air pollution, noise, green space and neurologic disease incidence: a population-based cohort study. Environ Health Glob Access Sci Source 2020;19:8. https://doi.org/10.1186/s12940-020-0565-4.

[24] Cantuaria ML, Waldorff FB, Wermuth L, Pedersen ER, Poulsen AH, Thacher JD, et al. Residential exposure to transportation noise in Denmark and incidence of dementia: national cohort study. BMJ 2021;374:n1954. https://doi.org/10.1136/bmj.n1954.
